# An evaluation of the hypolipidemic effect of an extract of Hibiscus Sabdariffa leaves in hyperlipidemic Indians: a double blind, placebo controlled trial

**DOI:** 10.1186/1472-6882-10-27

**Published:** 2010-06-17

**Authors:** Rebecca Kuriyan, Divya R Kumar, Rajendran R, Anura V Kurpad

**Affiliations:** 1 Division of Nutrition, St. John's Research Institute, St. John's National Academy of Health Sciences, Bangalore 560034, India; 2 Green Chem, Domlur, Bangalore, India

## Abstract

**Background:**

Hibiscus sabdariffa is used regularly in folk medicine to treat various conditions.

**Methods:**

The study was a double blind, placebo controlled, randomized trial. Sixty subjects with serum LDL values in the range of 130-190 mg/dl and with no history of coronary heart disease were randomized into experimental and placebo groups. The experimental group received 1 gm of the extract for 90 days while the placebo received a similar amount of maltodextrin in addition to dietary and physical activity advice for the control of their blood lipids. Anthropometry, blood biochemistry, dietary and physical activity were assessed at baseline, day 45 and day 90.

**Results:**

While body weight, serum LDL cholesterol and triglyceride levels decreased in both groups, there were no significant differences between the experimental and placebo group.

**Conclusions:**

It is likely that the observed effects were as a result of the patients following the standard dietary and physical activity advice. At a dose of 1 gm/day, hibiscus sabdariffa leaf extract did not appear to have a blood lipid lowering effect.

**Trial Registration:**

REFCTRI2009000472

## Background

In India, non-communicable diseases such as cardiovascular disease and cancer are emerging as major causes of death [1]. Hypercholesterolemia, smoking, hypertension, glucose tolerance, obesity and physical activity are some of the major modifiable risk factors for coronary heart disease. The non-pharmacological treatment for hypercholesterolemia includes dietary modification, weight loss or control, aerobic exercise, reduced alcohol consumption and cessation of smoking. A plant-based diet rich in fruit, vegetables and legumes and low in saturated fat along with regular exercise is the standard prescription for individuals with elevated risk of cardiovascular disease. Additionally, some herbs have been thought to help reduce hyperlipidemia, abnormal tendency to form blood clots, impaired blood flow or other cardiovascular problems [2]. Herbal medicine is based on the premise that plants contain natural substances that can promote health and alleviate illness [2]. Herbs refer to not only the herbaceous plants but also to bark, roots, leaves, seeds, flowers and fruits of trees, shrubs and woody vines.

*Hibiscus sabdariffa *(Linn) (family Malvaceae), is an annual dicotyledonous herbaceous shrub popularly known as 'Gongura' in Hindi or 'Pulicha Keerai' in Tamil. This plant is well known in Asia and Africa and is commonly used to make jellies, jams and beverages. In folk medicine, it has been used to treat hypertension [3], inflammatory disease [4] and cancer [5]. The flowers of Hibiscus sabdariffa contain anthocyanins, flavonoids and polyphenols [6]. Studies have highlighted the role of polyphenolic acid, flavonoids and anthocyanins that may act as antioxidants or have other mechanisms contributing to the cardio protective actions [7,8].

Animal studies have demonstrated that the extract of Hibiscus sabdariffa inhibited low-density oxidation in vitro and decreased serum cholesterol levels in cholesterol-fed rats and rabbits [9,10]. The only study in humans by Lin et al., 2007 [11] demonstrated that 2 capsules of hibiscus sabdariffa extract (1 g), given 3 times a day (for a total of 3 g/day), significantly lowered serum cholesterol in hypercholesterolemic patients. However, this study was done for a short duration (4 weeks) and importantly, it was not clear whether the body weight or dietary habits changed during the course of the treatment. Thus, the aim of the present study was to carefully evaluate the efficacy of an aqueous extract of Hibiscus sabdariffa leaves on the blood lipid levels of patients with hyperlipidemia requiring only dietary or lifestyle management.

## Methods

### Subjects

The study was a double blind, placebo controlled, randomized trial. Sixty subjects with serum LDL values in the range of 130-190 mg/dl and with no history of coronary heart disease were recruited into the study. Three subjects dropped out of the study and 57 subjects completed the study, such that there were 29 subjects in the placebo group and 28 subjects in the experimental group. The subjects (31 male and 26 female subjects) were aged between 35 and 60 years.

Exclusion criteria were the presence of any chronic disease and the concurrent use of any medication for the control of lipid levels. The subjects were recruited from the Nutrition and Lifestyle Management Clinic, St. John's Medical College Hospital, Bangalore. After recruitment, the subjects were randomly assigned into the placebo or experimental group. The study was approved by the institutional ethical review committee of St. John's Medical College and informed consent was obtained from the subjects.

### Experimental Protocol

The extract of Hibiscus Sabdariffa was obtained from the plant Hibiscus Sabdariffa Linn, which was cultivated by Green Chem, Bangalore, India. This is a common edible plant grown in Tamil Nadu and Andhra Pradesh. The leaves were harvested, sun dried for 3-4 days, powdered and stored under dry conditions. The extract was prepared using a Hydro Alcoholic mixture (50% Ethyl alcohol and 50% water) and heating at 70-75°C for about 4 hours in a closed system by re pumping the extract to the herb bed. This process was repeated twice. The stability of hibiscus extract was tested by heating it at 100-105°C for 1 hr, 2 hr, 4 hr, 8 hr and 16 hours and checking the product profile and estimating the phenolic compound content. It was observed that there was no change in the phenolic compound content and no change in the product profile. After heating, the extracts were then transferred to a thin film evaporator. It was concentrated initially to remove the solvent and then concentrated under vacuum to remove water at temperature less than 60°C. The product was purified and dried using a spray drier unit. The product was then powdered in a multimill to a fine mesh size. The powder was then sieved using a sifter to make uniform particle size, mixed in a blender to make a uniform and homogenous mixture. It was then packed in food grade, virgin, double polythene bags. Twelve kg of Hibiscus sabdariffa dried leaves or 60 kg of fresh leaves produced 1 g of the extract. Maltodextrin capsules (1 g/day) were used as placebo, and both capsules were prepared by Green Chem, Bangalore, India. Prior to the intervention, the subjects underwent baseline investigations which included anthropometric, biochemical, dietary and physical activity assessment. The extract was administered as two 500 mg capsules daily (1 g/day) for 90 days, during which the subjects reported weekly to the Nutrition Clinic to record their body weight, collect their weekly capsule supply and report adverse events, if any. The compliance of the subjects to the ingestion of capsules was documented every week when they reported to the Nutrition Clinic. The subjects were provided with a capsule calendar on which they were required to tick mark boxes relating to the daily intake of capsules and also to note down any missed capsule. The calendar and the 'missed' pill count were monitored every week. All the study subjects were provided with standard dietary and physical activity advice for the control of their lipid levels using the National Cholesterol Education programme (NCEP) guidelines. In case of overweight patients, advice was provided to achieve moderate weight loss of about 5%. The standard advice also included regular physical activity, with dietary strategies to increase dietary fiber (legume, fruits and vegetables) and decrease intake of fat and saturated fat. The compliance of the subjects to the prescribed diet and physical activity was assessed weekly by asking the subjects to rate their compliance on a scale of 0-100%.

### Anthropometric measurements

Body weight, height, skinfold thickness and mid-arm, waist and hip circumferences measurements were standardized [12]. Skinfold measurements in triplicates were carried out using Holtain skinfold calipers, at four sites (i.e.) biceps, triceps, subscapular and suprailiac. The average sum of four skinfold measurements were used to compute body density using the age and gender specific equation [13] and percent body fat was derived from body density [14]. These equations were previously validated in a group of Indian men and women [15]. The measurements were taken at baseline and repeated at day 45 and day 90 of the intervention period.

### Biochemical measurements

Fasting and post prandial blood glucose (collected 2 hours after breakfast), hemoglobin and lipid profile were measured at baseline, day 45 and day 90 of the study period. The blood glucose, triglyceride, total and HDL cholesterol were estimated by spectrophotometric assays on automated clinical chemistry analyzer -Dimension RxL (Dade Behring, Newark, USA), while LDL cholesterol was calculated from primary measurements using the empirical formula of Friedewald equation [16]. All assays were calibrated by use of Dade Dimension human calibrator (Dade Behring Inc, Newark, USA). The analytical coefficient of variation (inter-assay) for total cholesterol, triglycerides and HDL cholesterol were 4.1%, 4.7% and 4.1% respectively, while it was 2.6% for glucose.

### Dietary and Physical activity assessment

A 24 hr recall at baseline, repeated at day 45 and day 90 of the intervention period was carried out to assess the dietary intake of the subjects. The data from the dietary recall was used to arrive at estimates of daily nutrient intake from standard recipes, using the published food composition databases [17,18]. The routine physical activity pattern of the subjects was assessed using a questionnaire at baseline, day 45 and day 90 of study period. The questionnaire requested details regarding the time spent by patients in different activities such as occupation, travel, household and leisure activities. This allowed for an assessment of time spent in sedentary, moderately active or vigorously active domains of activity during the day, and any changes thereof, during the experiment.

### Statistical analyses

The data are presented as Mean ± SD. An independent 't' test analysis was performed to ascertain whether significant differences existed between the anthropometric and biochemical parameters of the subjects in the experimental and placebo group at baseline. A repeated measure ANOVA with group as a factor was performed to assess the change over time in the anthropometric, biochemical and food intake parameters between the two groups. The repeated measure ANOVA was then used to assess for significant differences between the various time points in the subjects of both groups independently. The significance level was set at p < 0.05.

## Results

Table 1 summarizes the profile of the subjects in the experimental and placebo groups at baseline. The mean age of the subjects in the experimental group was 45.7 ± 7.1 years and 46.2 ± 6.1 years in the placebo group. There were no significant differences in the mean age, weight, percent body fat, hemoglobin (Hb), fasting blood glucose, post prandial blood glucose and lipid profile between the experimental and placebo groups (Table 1).

**Table 1:** Profile of subjects at baseline

Parameter	Group	Mean ± SD	P value
Age (yrs)	Experimental	45.7 ± 7.1	0.19
	Placebo	46.2 ± 6.1	

Body weight (kg)	Experimental	63.4 ± 8.7	0.09
	Placebo	68.4 ± 9.7	

% Fat (#)	Experimental	24.8 ± 7.2	0.70
	Placebo	24.1 ± 7.2	

Hemoglobin (g %)	Experimental	14.7 ± 1.9	0.99
	Placebo	14.8 ± 2.2	

Fasting blood glucose (mg/dl)	Experimental	93.7 ± 9.3	0.09
	Placebo	88.8 ± 9.6	

Post prandial blood glucose (mg/dl)	Experimental	101.0 ± 26.3	0.55
	Placebo	97.2 ± 20.1	

Total cholesterol (mg/dl)	Experimental	207.2 ± 32.5	0.76
	Placebo	204.9 ± 24.0	

HDL cholesterol (mg/dl)	Experimental	42.0 ± 9.4	0.68
	Placebo	40.9 ± 9.2	

LDL cholesterol (mg/dl)	Experimental	155.3 ± 13.9	0.37
	Placebo	151.9 ± 14.9	

TG (mg/dl)	Experimental	159.9 ± 90.9	0.46
	Placebo	144.6 ± 60.1	

Mean ± Standard deviation (SD). # - Calculated from the sum of four skinfold measurements and applying the formulae of Durnin and Womersley (1974). No significant differences were observed in any of the parameters between the subjects of the two groups (independent 't' test)

The anthropometric parameters of the subjects in the experimental and placebo groups at various time points of the study are summarized in Table 2. There were no significant differences observed in the change of body weight, Body Mass Index (BMI), waist circumference, hip circumference and percent body fat over time between the two groups (repeated measure ANOVA). At the end of the study period, small but significant decreases in body weight and BMI were observed in both the experimental and placebo group, when compared to baseline parameters. There were no changes in the other parameters within the groups when compared to baseline parameters.

**Table 2:** Anthropometric parameters of the subjects at baseline, day 45 and day 90 of the study

Parameter	Baseline	Day 45	Day 90	F value	P value
Body weight (kg)					
Experimental	63.4 ± 8.7	63.0 ± 8.7	62.6 ± 8.6*	0.17	0.84
Placebo	68.4 ± 9.7	67.8 ± 9.4	67.6 ± 9.4*		

Body mass index (kg/m^2^)					
Experimental	25.2 ± 3.3	24.9 ± 3.3	24.8 ± 3.2*	0.27	0.73
Placebo	26.3 ± 2.8	26.0 ± 2.8	25.9 ± 3.0*		

Mid arm circumference (cm)					
Experimental	29.8 ± 3.1	29.9 ± 3.0	30.0 ± 3.4	1.8	0.16
Placebo	30.4 ± 3.1	30.9 ± 4.9	29.9 ± 3.6		

Waist circumference (cm)					
Experimental	87.3 ± 8.6	87.3 ± 8.7	87.2 ± 8.5	1.58	0.21
Placebo	91.9 ± 7.0	91.3 ± 6.4	91.6 ± 6.6		

Hip circumference(cm)					
Experimental	96.6 ± 6.5	96.6 ± 6.0	96.3 ± 5.9	0.43	0.65
Placebo	97.8 ± 7.1	97.7 ± 6.6	97.7 ± 6.4		

Percent Fat (%) #					
Experimental	24.8 ± 7.2	23.4 ± 5.9	23.4 ± 5.9	2.7	0.07
Placebo	24.0 ± 7.2	24.3 ± 7.6	24.7 ± 7.9		

Mean ± SD; # - Calculated from the sum of four skinfold measurements and applying the formulae of Durnin and Womersley (1974). n = 29 in placebo & n = 28 in experimental group

No significant interaction between time points and group (repeated measure ANOVA with group as between subject factor). *-Significant difference observed between time points within each group (repeated measure ANOVA)

There were no significant interaction effect observed between time and the group (repeated measure ANOVA) in any of the biochemical parameters (Table 3). There was a significant 18% (28 mg/dl) decrease in the LDL levels of the experimental group at day 90 when compared to the baseline, while in the placebo group there was a significant 12% (19 mg/dl) decrease (repeated measures, ANOVA). The serum triglycerides of the experimental group had a significant 10% reduction at day 90 (143.3 ± 73.9 mg/dl) when compared to baseline (159.9 ± 90.6 mg/dl), while the placebo group did not show any significant change. There were no significant changes observed in total cholesterol, HDL, Fasting and Post Prandial blood sugars of the experimental and placebo group at end of the study when compared to the baseline.

**Table 3:** Biochemical parameters of the subjects at baseline, day 45 and day 90 of the study

Parameter	Baseline	Day 45	Day 90	F value	P value
Total Cholesterol (mg/dl)					
Experimental	207.1 ± 32.5	208.2 ± 32.3	198.5 ± 33.2	2.53	0.09
Placebo	204.9 ± 23.9	202.6 ± 24.3	198.6 ± 21.2		

HDL Cholesterol (mg/dl)					
Experimental	42.0 ± 9.4	41.6 ± 9.5	42.6 ± 8.9	0.32	0.73
Placebo	40.9 ± 9.2	39.6 ± 9.0	41.2 ± 9.3		

LDL cholesterol (mg/dl)					
Experimental	155.3 ± 13.9	145.2 ± 25.6	127.6 ± 24.1*	1.0	0.36
Placebo	150.7 ± 14.3	142.4 ± 24.8	132.0 ± 17.1*		

Serum Triglycerides (mg/dl)					
Experimental	159.9 ± 90.6	134.6 ± 56.2	143.3 ± 73.9*	0.07	0.92
Placebo	144.6 ± 60.1	134.6 ± 56.2	130.6 ± 51.2		

Fasting Blood Glucose (mg/dl)					
Experimental	93.7 ± 9.3	89.7 ± 13.7	93.1 ± 9.9	2.5	0.09
Placebo	88.8 ± 9.6	91.4 ± 13.7	92.2 ± 8.5		

Post Prandial Blood Glucose (mg/dl)					
Experimental	100.9 ± 26.3	110.7 ± 29.6	110.6 ± 21.6	1.2	0.32
Placebo	97.2 ± 20.9	104.4 ± 17.7	113.4 ± 18.7		

Mean ± SD. n = 28 in experimental group and 29 in Placebo group. No significant interaction between time points and group (repeated measure ANOVA with group as between subject factor). *-Significant difference observed between time points within each group (repeated measure ANOVA).

The food intake data of the subjects are summarized in Table 4. There were no significant differences observed in the change of energy, protein, carbohydrate, fat, protein, and cholesterol intake over time between the two groups (repeated measure ANOVA). The energy intake was significantly reduced by about 12% (214 kcal/d) in the experimental group, while there was a significant decrease of about 9% (189 kcal/d) in the placebo group at day 90, when compared to baseline. While there was trend towards a decrease in the protein, carbohydrate, fat and cholesterol intakes at day 90 in both the experimental and placebo group when compared to baseline values, these changes were not significant.

**Table 4:** Food intake data of the subjects at baseline, day 45 and day 90 of the study

Parameter	Baseline	Day 45	Day 90	F value	P value
Energy (kcal/day)					
Experimental	1845.2 ± 803.9	1619.4 ± 510.6	1631.6 ± 516.5*	0.10	0.82
Placebo	2018.8 ± 704.1	1762.9 ± 396.5	1829.9 ± 543.3*		

Protein (g/day)					
Experimental	52.0 ± 20.6	46.9 ± 12.8	45.9 ± 12.8	0.14	0.82
Placebo	59.6 ± 21.6	53.4 ± 10.5	54.8 ± 16.8		

Fat (g/day)					
Experimental	37.8 ± 16.5	38.0 ± 16.5	39.1 ± 17.7	0.47	0.62
Placebo	50.6 ± 31.5	45.2 ± 16.8	49.8 ± 26.8		

Carbohydrate (g/day)					
Experimental	287.1 ± 100.1	252.8 ± 63.2	255.3 ± 65.1	0.20	0.80
Placebo	331.3 ± 110.0	285.5 ± 70.3	290.5 ± 76.2		

Cholesterol (mg/day)					
Experimental	96.6 ± 117.5	89.0 ± 104.3	68.7 ± 88.4	1.9	0.15
Placebo	89.1 ± 104.9	60.0 ± 37.7	76.3 ± 88.9		

Mean ± SD. n = 28 in experimental group 29 in placebo

No significant interaction between time points and group (repeated measure ANOVA with group as between subjects factor)

*- Significant difference observed between time points for each group (repeated measure ANOVA)

The mean reported compliance of the subjects in the experimental group to their prescribed diet was 83% (65-95%) and 84% (55-95%) to prescribed physical activity, while in the placebo group it was 85% (60-90) to prescribed diet and 80% (50-98%) to prescribed physical activity. The physical activity pattern of both the experimental and placebo group did not change during the study.

There were no serious adverse events reported by the subjects of the present study and events were limited to mild gastrointestinal symptoms such as abdominal distention, flatulence and epigastric pain during the first week of supplementation. These symptoms were present in both the experimental (29%) and placebo group (27%) of subjects. The symptoms subsided within a week in all subjects.

## Discussion

The mechanism by which plants and their active dietary constituents play a role in the prevention of many chronic diseases is not clear and still controversial. Recent research has focused on various herbs that possess hypolipidemic, hypoglycemic, antiplatelet, antitumor properties that may be useful adjuncts in helping in reducing the risk of chronic diseases. Hibiscus sabdariffa is a traditional Chinese rose tea, widely cultivated in tropical areas and is used effectively in folk medicine. The chemical constituents reported in Hibiscus sabdariifa are phenolic compounds, anthocyanins, flavonoids, protocatechuic acid, Vitamin C and carotenoid [19]. Studies on the cholesterol lowering effect of Hibiscus sabdariffa have been conducted on animals [9,10] and humans [11]. The present study was aimed at carefully evaluating the lipid lowering effects of Hibiscus sabdariffa leaves in patients with elevated lipid levels.

There were no significant differences observed in the change in the lipid values between the subjects of the experimental and the placebo group. The dose of the extract in the present study was 1 g/day (provided as 2 capsules a day). The only other published study on humans [11] demonstrated that the consumption of hibiscus flower extract at a dose of 2 capsules (1 g) with every meal (for a total of 3 g/d) for 1 month significantly lowered the serum cholesterol levels. We had chosen the lower dose of the aqueous extract of Hibiscus leaves based on anecdotal references and personal discussions with local ayurvedic practitioners on their daily dose used for treatment. It is possible that the dose provided in this study of the hibiscus leaves is not adequate. Additionally, the sample size calculated based on the previous study was 38 in each group, but since this was conducted as a pilot study, we chose a sample size of 30 in each group.

While there were no significant differences observed between groups, the LDL cholesterol (18%) and triglycerides (10%) of the experimental group and the LDL levels of the placebo group (12%) were significantly reduced at day 90 when compared to baseline values. Additionally, small but significant reductions in body weight and body mass index were seen in both the group of subjects. Both the experimental and placebo group were provided with dietary and lifestyle advice for the control of their lipid levels and the reported compliance to the advice was good in both group (~83%). While the dietary data of the present study showed that the energy intakes of both groups were significantly lower at day 90 when compared to the baseline intake, it must be remembered that dietary records are known to underestimate food intakes, with individuals having greater weight and BMI being prone systematically to under reporting [20,21].

Lifestyle modifications with emphasis on normalization of body weight and healthy patterns of dietary intake and physical activity are the cornerstone of treatment of individuals identified as having high lipid levels. Individualized dietary advice for reducing blood cholesterol levels have shown to be modestly effective in free-living subjects; for eg diets low in saturated fat and cholesterol can lower LDL cholesterol by 11 to 15% [22]. Physical activity benefits most of the artherosclerotic risk factors and regular physical activity has been shown to reduce LDL cholesterol and triglycerides [23,24]

## Conclusion

Thus the present study suggests that at a dose of 1 g/day, hibiscus sabdariffa leaf extract did not seem to have a specific lipid lowering effect over and above the effect of standard dietary and lifestyle advice. It is possible that the positive effects seen in body weight, BMI, LDL cholesterol and serum triglycerides may have been due to the changes the subjects made in their food intake and physical activity during the period of experimental observation.

## Competing interests

We would like to mention that one of the authors, Mr. Rajendran had supplied the capsules for the study. However he was not involved in the planning or data analysis of the study.

## Authors' contributions

RK made substantial contributions to conception, design, and acquisition of data, analysis and interpretation of data and drafted the manuscript. DR was involved with the acquisition of data and running of the study. RR participated in the design of study and drafted the manuscript. AVK was involved in the conception and design of study, revised the manuscript critically for important intellectual content. The authors have all read and approved the final manuscript.

## Pre-publication history

The pre-publication history for this paper can be accessed here:

http://www.biomedcentral.com/1472-6882/10/27/prepub
